# Physical Cues in the Microenvironment Regulate Stemness-Dependent Homing of Breast Cancer Cells

**DOI:** 10.3390/cancers12082176

**Published:** 2020-08-05

**Authors:** Hsueh-Yao Chu, Yin-Ju Chen, Chun-Jieh Hsu, Yang-Wei Liu, Jeng-Fong Chiou, Long-Sheng Lu, Fan-Gang Tseng

**Affiliations:** 1 Department of Engineering and System Science, National Tsing Hua University, Hsinchu 30013, Taiwan; kecokoyo@gmail.com (H.-Y.C.); jack013626@gmail.com (C.-J.H.); Ywliu218@gmail.com (Y.-W.L.); 2 Department of Radiation Oncology, Taipei Medical University Hospital, Taipei 11031, Taiwan; yjchen1113@gmail.com (Y.-J.C.); solomanc@tmu.edu.tw (J.-F.C.); 3 Graduate Institute of Biomedical Materials and Tissue Engineering, College of Biomedical Engineering, Taipei Medical University, Taipei 11031, Taiwan; 4 International Ph.D. Program in Biomedical Engineering, College of Biomedical Engineering, Taipei Medical University, Taipei 11031, Taiwan; 5 Department of Radiology, School of Medicine, College of Medicine, Taipei Medical University, Taipei 11031, Taiwan; 6 Taipei Cancer Center, Taipei Medical University, Taipei 11031, Taiwan; 7 International Ph.D. Program for Cell Therapy and Regeneration Medicine, College of Medicine, Taipei Medical University, Taipei 11031, Taiwan; 8 Department of Engineering and System Science, Frontier Research Center on Fundamental and Applied Sciences of Matters, National Tsing-Hua University, Hsinchu 30013, Taiwan; 9 Research Center for Applied Sciences, Academia Sinica, No. 128, Sec. 2, Academia Rd., Nankang, Taipei 11529, Taiwan

**Keywords:** extracellular matrix, stiffness, nanotopography, cell homing, tumor microenvironment, hydrogel

## Abstract

Tissue-specific microenvironmental factors contribute to the targeting preferences of metastatic cancers. However, the physical attributes of the premetastatic microenvironment are not yet fully characterized. In this research, we develop a transwell-based alginate hydrogel (TAH) model to study how permeability, stiffness, and roughness of a hanging alginate hydrogel regulate breast cancer cell homing. In this model, a layer of physically characterized alginate hydrogel is formed at the bottom of a transwell insert, which is placed into a matching culture well with an adherent monolayer of breast cancer cells. We found that breast cancer cells dissociate from the monolayer and home to the TAH for continual growth. The process is facilitated by the presence of rich serum in the upper chamber, the increased stiffness of the gel, as well as its surface roughness. This model is able to support the homing ability of MCF-7 and MDA-MB-231 cells drifting across the vertical distance in the culture medium. Cells homing to the TAH display stemness phenotype morphologically and biochemically. Taken together, these findings suggest that permeability, stiffness, and roughness are important physical factors to regulate breast cancer homing to a premetastatic microenvironment.

## 1. Introduction

Cancer is the second leading cause of human death worldwide [1], and its metastasis is responsible for up to 90% of cancer-related deaths [2,3]. Metastatic tumor cells can adopt a variety of migration strategies to home to distant organs, and their interaction with extracellular matrix is under active investigation [4,5,6,7]. Previous studies have shown that the structural properties of the extracellular matrix (ECM) in the tumor microenvironment promote cell migration via biochemical and biophysical interactions [8,9,10,11,12,13,14]. The motility, division, signaling, and differentiation of tumor cells are affected by the ECM, which enhance tumor metastasis [15,16,17,18]. In addition to paracrine signaling, physical properties like mechanosensing may contribute to such complex interactions. Biophysical properties including stiffness, porosity, density, and surface topology [12,19,20,21,22] have all been implicated in cellular signals that trigger migratory capacity. Furthermore, an intrinsic cell state, such as the degree of differentiation, may be fine-tuned by differential mechanosensing thresholds, which contribute to tumor metastasis and growth [23,24,25]. This concept is close to a general observation that the higher stiffness of ECM is prone to the development of cancer metastasis [23,26,27,28,29,30]. It is also known that cancer is a stem cell-based disease. The growth of a solid tumor is supported by cancer stem cells (CSCs), which are characterized as self-renewing, and having unlimited proliferation and drug resistance [31,32,33,34]. The main challenge in the study of CSCs is the isolation and propagation because only a small amount of CSC is found in solid tumors. For example, in breast cancer, only about 2% of cells are found to be CSCs [35]. In order to isolate CSCs efficiently, serum-free medium and fluorescence activated cell sorting (FACS) are widely used. However, the cost and labor-intensive protocols used in both methods restrict the development of CSC research and the yield of viable cells are quite low. Consequently, to establish a new and economical screening method to isolate and propagate CSCs in vitro is a crucial need [36,37]. 

A model system with controllable ECM parameters is essential for biophysical stimulation guiding cell invasion and migration. This model can be used in the potential therapeutic drug screening and other clinical applications. Although the two-dimensional (2D) cell culture system has become a typical testing standard used in exploring in vitro cellular responses to the biophysical stimulation [38,39,40], it still has some physiological limitations, especially in morphology and biochemistry. Cells cultured in a 2D environment can not precisely reflect the real situation in vivo. For this reason, developing a three-dimensional (3D) cell culture system is crucial in cancer research, such as exploring the cell motility, proliferation, tissue formation, and metastasis processes. These phenomena have been proven to be regulated by mechanical interactions with the surrounding microenvironment [41,42,43,44,45,46,47]. Moreover, current metastasis research in different types of cancer heavily relies on animal models where the human–mouse xenograft is mostly adopted. Hence, a simple, efficient, and well-recognized metastasis model is urgently needed for not only avoiding the unreliable assessment of cancer biology, but also for demonstrating a standardized and realistic prototype for the investigating of disease mechanisms, drug efficacy, and cell characterization [48,49,50,51]. The cell migration behavior in a 3D system includes three steps: detaching from the original sites, entering the medium, and finding an appropriate site (homing) for seeding and proliferation. However, until now, although comprehensive 3D in vitro models have been investigated to better simulate physiological conditions in humans, no one can genuinely mimic these homing processes successfully. The emergence of these models sheds light on many advantages of 3D systems including convincible reproducibility, accurate environmental controls over culturing, and the incorporation of other cells for simulating the growth conditions of tumors in vivo [52,53,54]. Also, they offer alternative systematic and quantitative approaches to mouse models.

Hydrogel is a biological compatible material with efficient oxygen and nutrient transportation and decent biodegradability [55,56,57]. With an adjustable curing procedures using bivalent ions, hydrogel can precisely simulate properties like structural formation and the mechanical strength of human tissues or organs with excellent permeability. Due to its easy handling, it is one of the most popular materials for tissue engineering. Fundamental research studies of the model based on hydrogel for 3D tissue engineering have shown that hydrogel-derived modules can highly mimic the complex ECM environments and provide flexible capability to modify their physical and biochemical properties [43,44,46,58]. Previous studies have already shown that some physical properties like the stiffness of hydrogel matrix is related to the process of cancer cell metastasis [41,59,60,61]. Also, the topological structure of hydrogel is involved in the recruitment, maintenance, and proliferation of cancer cells [29,62,63]. According to these characteristics, a 3D model built with hydrogel is the finest method for rebuilding tumor microenvironment in vitro, which can be customized to investigate the interaction between cancer cells and ECM [41,44,64].

**Figure 1:**
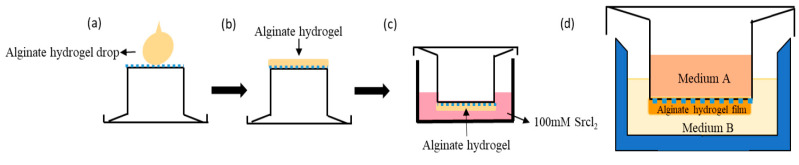
Schematic of transwell-based alginate hydrogel (TAH) apparatus and principle of curing. (**a**) Apply 4% sodium alginate on the membrane of transwell. (**b**) Spread alginate hydrogel equally. (**c**) Invert the transwell in a well with 100 mM SrCl_2_ for curing overnight. (**d**) Assemble a TAH platform by the transwell and a 24-well plate containing breast cancer cells and the medium.

In this study, a platform combining “ transwell insert” and sodium alginate hydrogel films was developed, which is suitable for studying the homing process of breast cancer cells in a 3D environment [65,66], as shown in Figure 1c. Based on this setup, the engineered hydrogel film was able to mimic the ECM microenvironment, in which the stiffness, porosity, and surface topology of sodium alginate can be adjusted independently to one another [61,67,68]. Two breast cancer cell lines were used in this research for the investigation of the relationship between cell homing behavior and ECM microenvironments.

Our results identify the homing cues of breast cancer, which are listed as follows: firstly, adherent cancer cells are able to detach from the original growth site and home to a proper microenvironment for further colonization and proliferation; secondly, stiffness and surface topography are crucial parameters for cell homing, which can be adjusted easily by physical treatments without any additional chemical modification of alginate; the last and the most important cue is that homing cells display stemness features in morphology and related gene expression profiles, which reflects their metastatic potential like CSCs in humans. In short, our TAH platform is the first model that can represent the closest homing behavior of cancer cells in vitro of that in human conditions. It will not only make some contributions in cancer biology for measuring metastasis capacity in vitro, but also become a novel platform for the development of anti-tumor drugs and therapies against metastatic cancer or tumor microenvironments.

## 2. Results

### 2.1. Manufacturing Process

#### 2.1.1. Manufacturing of Transwell Alginate Hydrogel-TAH

The manufacturing process of transwell-based alginate hydrogel (TAH) was carried out with a combination of alginate hydrogel and a 24-well plate with transwell inserts containing porous polycarbonate membrane (3.0 μm in diameter). Fifty microliters of 2%, 4%, and 6% (*w*/*v*) sodium alginate were applied on the membrane (Figure 1a) with inverted positions then whole transwell inserts (SPL Life Sciences Co., Ltd.; Figure 1b) were soaked in the solution of 100 mM SrCl_2_ for at least four hours to ensure that the TAH was totally solidified (Figure 1c). The thickness of the hydrogel film was controlled by the volume of alginate solution. The completed TAH was washed with 5 mM SrCl_2_-PBS solution three times and sterilized by UV light exposure overnight for further experiments. For different purposes, the medium containing specific growth factors or inhibitors and cells isolated from specific organs could be placed or seeded in the top space of the hydrogel film (medium A), while the growth medium of breast cancer cells was retained in the well of the plate (medium B), as shown in Figure 1d. 

#### 2.1.2. Developing a Biophysical Cell Homing Model with Alginate Hydrogel

**Figure 2:**
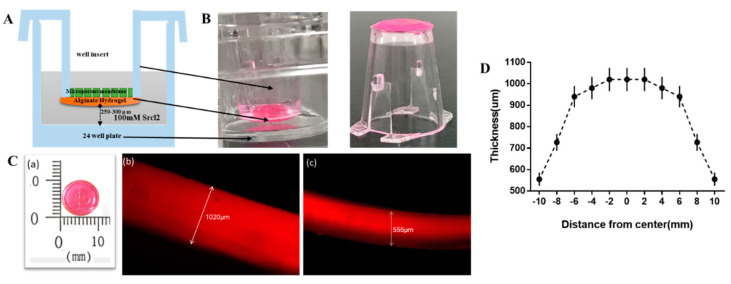
Preparation of experimental materials (**A**) Schematic diagram of transwell-based alginate hydrogel (TAH) homing model. (**B**) For the purpose of scope measurement, alginate hydrogel was dyed with Rhodamine 6G and the appearance of TAH, and (**C**) The top view (a) of actual size of TAH and side views (b and c) using fluorescence microscopy for the measurement of thickness. (**D**) The thickness distribution of the 4% hydrogel film constructed in a TAH model.

Testing different physical conditions in alginate hydrogel may help us understand how certain cancer cells can migrate spontaneously in specific directions. In this study, we used a transwell-based alginate hydrogel (TAH) model which develops a pre-metastatic niche (PMN) to simulate the phenomenon of tumor cell homing (Figure 2A). The transwell insert was compatible with the conventional 24-well cell culture plates and could be simply placed into any well. Also, the transwell insert had extremely low fluid shear stress on the surface of the 24-well culture plate. It could be used to predict the gradient effect of tissue explants. The distance from alginate hydrogel to the bottom of the well was above 250 μm (Figure 2B). TAH films could be separated from the transwell inserts easily by tweezers, and they were in circular shape with diameters in about 10 mm (Figure 2C-a) in appearance. Also, the thickness of center point was about 1020 μm (Figure 2C-b) and the edge was about 555 μm (Figure 2C-c). Due to the high-water content of alginate hydrogel, the film was thicker in the center led by surface tension when curing by the hanging drop method. Figure 2D shows the distribution of thickness of hydrogel films forming in TAH model, which can be roughly divided into two parts: the thickness of outer ring area (8 to 10 mm from the center) is around 555 to 727 μm and that of inner ring (within 6 mm from the center) is between 940 to 1020 μm.

### 2.2. Biophysical Cues and Cell Behaviors

#### 2.2.1. TAH as a Molecular Sieving Substrate to Attract Cancer Cells via Growth Factors

**Figure 3:**
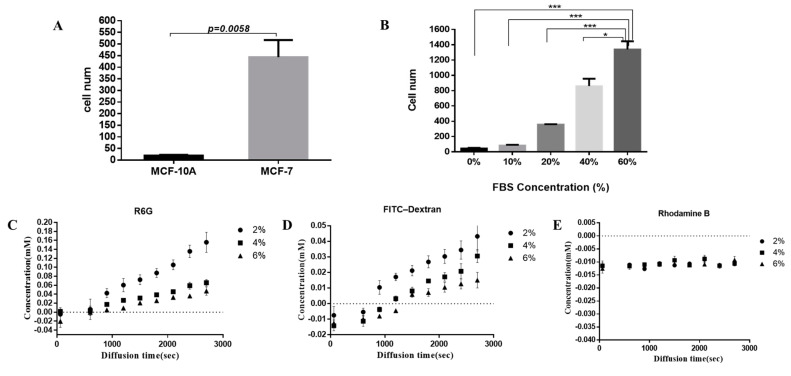
(**A**) The number of homing cells of MCF-10A and MCF-7 in 4% TAH. Values shown are mean ± SD of three independent experiments. *p* value = 0.0058. (**B**)The number of MDA-MB-231 cells homing to TAH surface with different concentrations of fetal bovine serum (FBS). Experiments were performed in triplicate. A two-tailed unpaired ANOVA with Tukey’s HSD post hoc test. * *p* < 0.05, *** *p* < 0.001. (**C**–**E**) The permeability was measured of R6G, FITC-Dextran and Rhodamine B in 2 to 6% (*w*/*v*) TAH. The diffusion rate was determined by dynamic measuring of concentrations of tested substance at different time from 0 to 2800 s.

To assess the impact of TAH characteristics with breast cancer cells, we performed a homing test on normal breast epithelial cell line MCF-10A. MCF-10A and MCF-7 were tested for homing efficiency in the 4% TAH for 72 h incubation. The normal human breast cell line MCF-10A shows much lower (9- to 10-folds less) homing than breast cancer cell MCF-7. It is noteworthy that a normal breast epithelial cell line MCF-10A only has minimal homing capability in TAH model as shown in Figure 3A. Therefore, the cell homing described in this manuscript is very likely a behavior specific to malignant cells; TAH is a potential model that reflects the types of cancer cell migration.

The physiological effects of bio-gels were not limited to their mechanical properties. They could also form selective barriers that controlled the exchange rate of molecules between different compartments. The culture medium containing 0%, 10%, 20%, 40%, and 60% of fetal bovine serum (FBS), respectively, was loaded into the top space of 4% alginate (*w*/*v*) membrane inserts. Then, each well in the homing test device was seeded with about 3 × 10^3^ of MDA-MB-231 cells. The calculation of the number of cells that attached to the alginate film after 24 h of incubation is described in Section 4.6. The tendency could be observed that the number of homing cell was increasing with the higher concentration of FBS in the different TAH groups. Comparing to the group of 10% FBS (*v*/*v*) in a common culturing condition, homing cells in the medium containing 60% FBS was about tenfold. This indicated that MDA-MB-231 cell migration and invasion were dose-dependent (*p* value = 0.0002), as shown in Figure 3B. The phenomenon proved that the permeable property in alginate hydrogel films could induce the cell to home, presumably because nutrients with high concentration could diffuse more into hydrogels. To further control and improve the diffusion rate and molecular transportation in hydrogels, the concentrations (*w*/*v*) of 2%, 4%, and 6% alginate hydrogel were prepared and three fluorophores were used to test the effect of gel concentration against solutes with different molecular weights. Rhodamine 6G (R6G), FITC-dextran, and Rhodamine B were used in this experiment. The molecular weight of Rhodamine 6G was 479, which was much less than 10,000 and able to pass through human microvascular. FITC-dextran was chosen to simulate the substance limited in microvascular with molecular weight of 20,000. Due to fact that the molecular selectivity of the microvascular was between 10,000 and 60,000, a substance like Rhodamine B with a molecular weight of 70,000 would be excluded from microvasculature. The diffusion rate of testing substances against different concentrations of hydrogel was observed and plotted with identical time intervals. The result showed that the pore size of hydrogels decreased with the increasing of alginate concentration, and the diffusion rate was lower in the tested materials with higher molecular weight. A previous study has demonstrated the increasing concentration of hydrogel is directly proportional to smaller pore size which leads to the reduction of relative diffusivity [69]. For this reason, the parameter of alginate hydrogel concentration was tested by the measuring the permeability of the fluorescent compound with different molecular weights in films composed of 2%, 4%, and 6% of hydrogel. Both R6G and dextran-FITC with molecular weight of 479 and 20,000 displayed a simple diffusion manner in 2% and 4% of hydrogel, and the latter compound showed slower diffusion rate in 6% hydrogel (Figure 3D). However, macromolecule Rhodamine B, which has a molecular weight of 70,000, was unable to penetrate any of them. The diffusion coefficients of compounds tested under different concentrations of alginate used in TAH were calculated according to Figure 3C to 3E respectively and are listed in Table 1.

#### 2.2.2. Controlling TAH Stiffness by Ethanol for Promoting Physicotactic Cell Migration

**Figure 4:**
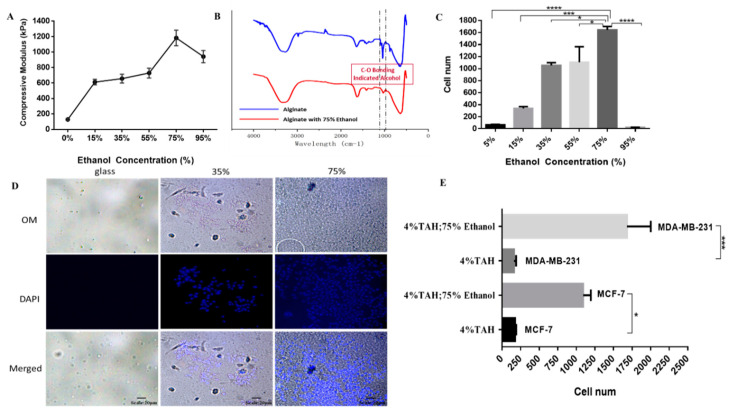
Biophysical parameters of TAH and cancer cell homing in this model. (**A**) The compressive modulus of TAH treated with different concentration of ethanol. (**B**) The FTIR absorption spectra of TAH (blue) and 75% ethanol-treated TAH (red). The range of hydroxyl group is from 1000 to 1100 cm^−1^, indicated by dotted lines. (**C**) The cell number of MDA-MB-231 homed to alginate hydrogel films pretreated with different concentrations of ethanol. Experiments were performed in triplicate. A two-tailed unpaired ANOVA with Tukey’s HSD post hoc test. * *p* < 0.05, *** *p* < 0.001, **** *p* < 0.0001. (**D**) Images of cell attached on hydrogel films treated with 35%, 75%, and 95% ethanol, respectively. Nuclei were stained by DAPI and observed by fluorescence microscopy with the magnification of 100× and shown in blue. Scale bar is 20 μm. (**E**) The cell number of attached MCF-7 & MDA-MB-231 on 4% alginate hydrogel films with or without ethanol treatment. This experiment was performed in triplicate. ANOVA with Tukey’s HSD post hoc test. * *p* < 0.05, *** *p* < 0.001.

The external stiffness of the tumor microenvironment determined the different behaviors of cancer cells, including the metastasis of tumor cells to the secondary colonization site. In order to construct various microenvironments to simulate the metastasis and explore the changes in the homing behavior of cancer cells caused by the different stiffness of alginate hydrogel, tests for the comparison of stiffness conversion of alginate hydrogel by the treatment of different concentrations of ethanol were carried out. Four percent alginate hydrogel films were immersed in 5%, 15%, 35%, 55%, and 75% (v/c) ethanol, respectively, for at least 12 h (overnight). Different concentrations of ethanol caused a difference in the stiffness of alginate hydrogel films, which were measured by a tensile compression static analyzer. The parameter of compression test was 2 cm in diameter and 1 cm in height of alginate hydrogel films which were compressed at a speed of 0.05 mm per second. Compression moduli under different conditions were compared, as shown in Figure 4A, and basically proportional to the ethanol concentrations. The highest modulus was found in the 75% ethanol treated hydrogel. Also, Fourier transform infrared spectroscopy (FTIR) was used to analyze the structure of functional groups, the covalent bonds, and the chemical constituents of alginate hydrogel films. Figure 4B shows that the concentration of ethanol was negatively correlated to the peak around the wavelength of 1000–1100 cm^−1^, which represented the carboxyl group derived by ethanol. No other redundant of functional groups were found. This indicated that the high concentration of ethanol caused strong dehydration of the gel, but the reaction was only restricted to lower the water content of hydrogel. However, since the alginate gel film was mostly composed by saline buffer containing lots of water, dehydration had a tremendous impact on its stiffness. 

In the homing model of TAH based on 24-well plate, every well was pre-seeded with 3 × 10^3^ of MDA-MB-231 breast cancer cells for at least 24 h. Then, the transwell insert attached with cured alginate hydrogel films pretreated with different concentrations of ethanol was placed in each well for another 24-h coincubation. The number of cell which homed from the surface of plate to hydrogel films was determined by using CellTiter-Glo^®^ (Promega, Southampton, UK) luminescent cell viability assay, as shown in Figure 4C. The cellular adhesion occurred the most in the group of 75% ethanol-treated alginate hydrogel, in which the average of cell number was 1643, followed by the group of 55% ethanol with an average of 1106 cells. Compared to the group containing 5% ethanol, the two conditions above had about 10 to 16 times (*p* < 0.0001) more cells migrate to alginate hydrogel films. We found that a higher concentration of ethanol pretreatment could lead to a higher dehydration impact which conferred to the higher stiffness of hydrogel. It could be speculated that the breast cancer cell MDA-MB-231 preferred the stiffer environment for its homing, and this was reflected in the number of cells attached to the hydrogel films. Figure 4D shows fluorescence microscope images (200×) of alginate hydrogel films treated with 35% and 75% ethanol, respectively. Cells attached on alginate hydrogel films were stained by DAPI to indicate the nucleus. The number of cells attached to the hydrogel films increased with the elevated concentration of ethanol pretreatment. Also, many clusters caused by cell aggregation could be observed in the group containing 75% ethanol. No cell homing or adhesion was observed in the control group which consisted of a 1.2 cm circular glass cover slip attached onto a transwell membrane. In short, 75% ethanol-pretreated alginate hydrogel film was the most favorable condition for breast cell migration. In order to confirm that the result was not due to biological bias, another breast cancer cell line, MCF-7, was also tested with identical conditions, as shown in Figure 4E. Both breast cancer cell lines showed similar trends in homing behavior and preferred hydrogel treated with 75% ethanol for their migration. The phenomenon of breast cancer cell homing in the TAH model was directly affected by biophysical parameters like stiffness of alginate hydrogel; comparing the MCF-7 (*p* value = 0.0203) and MDA-MB-231 (*p* value = 0.010) (with high metastatic ability) results indicated that the TAH was suitable to quantify the in vitro migration capacity of cancer cell.

#### 2.2.3. The Modification of Hydrogel Topography to Alter the Homing Behavior of Cells

**Figure 5:**
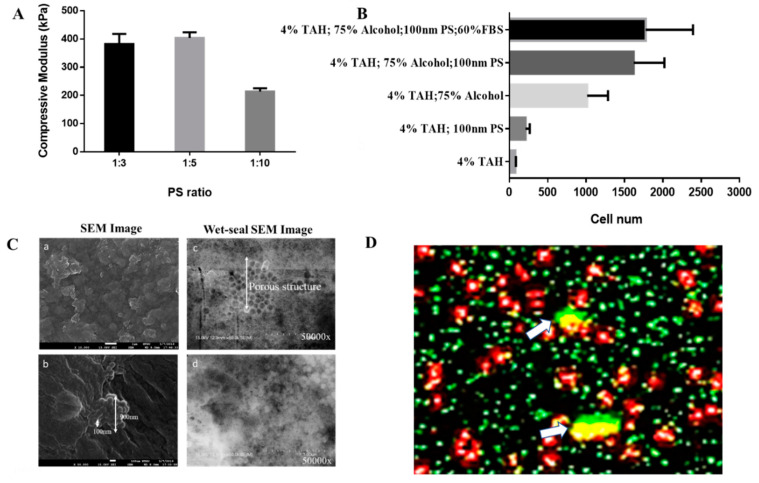
The effect of nanotopographical density on cell homing. (**A**) The compressive modulus of 4% alginate hydrogel mixed with PS nanoparticles in different ratios: 1:3, 1:5 and 1:10, where the ratio of 1:5 showed the greatest stiffness. (**B**) The number of MCF-7 cells attached on the surface of TAH pre-treated with different conditions. In the groups of TAH treated with 75% ethanol and PS nanoparticles resulted in better cell homing. (**C**) The surfaced structure of hydrogel doped by 100 nm PS nanoparticles was scrutinized by SEM and wet SEM. The magnification of (a) was 10,000× and others were 50,000×. (**D**) The overlaid image of MCF-7 cells attached on TAH/75% ethanol/PS beads was recorded by confocal microscopy. The ratio of total area of PS nanoparticles (green) to cell covering area (red) was 0.045.

Surface properties of substrates are crucial for metastasis, especially in roughness, which is proportional to cell adhesion and colonization. Polystyrene (PS) nanoparticle is a polymer that is commonly used in biomedical applications. To investigate the effects of surface topography of TAH in the adhesion and subsequent proliferation of breast cancer cells, MCF-7 was used in homing tests in the TAH model where the surface was pre-modified by PS nanoparticles. The mixing ratio of 100 nm PS nanoparticles to 4% alginate hydrogel was 1:3, 1:5, and 1:10 by volume. The highest compressive modulus was observed in the nanocomposite containing PS nanoparticles in the ratio of 1:5, as shown in Figure 5A. By modifying the mixing ratio of sodium alginate and inert nanoparticles, we obtained hydrogels with a range of mechanical stiffness. The surface structure of hydrogel after dehydration with 75% ethanol was observed by SEM (Figure 5C). The hydrogel containing PS nanoparticles (Figure 5C-a) was more prominent in columnar structures and rough surface. Alginate hydrogel films treated with both nanoparticles and ethanol were examined by SEM at 50,000× magnification. The surface structure showed not only many clusters formed by large PS nanoparticles spheres, but also many potholes and concaves, as shown in Figure 5C-b. All samples observed by electron microscopes above were dehydrated by ethanol, but alginate hydrogel was the material with extremely high-water content and its structure would be distorted after dehydration. Structural deformation would be problematic for the observation of the actual surface of the hydrogel. To conquer this issue, liquid SEM (Flow AOI) was introduced for the observation of liquid samples like alginate hydrogel. The sample preparation method was the same as the one used for SEM scanning. The surface of hydrogel without dehydration was observed, as shown in Figure 5C (c & d), in which many round and circular structures ranging in size from 30 to 70 nm in diameters could be found. They also represented some dark areas with honeycomb structures that were speculated as autogenic concaves formation when the hydrogel was cured by strontium ion. Furthermore, white particles/patches could be found randomly in different areas, and they were about 100 nm in diameter. They were PS nanoparticles or the formation of concaves after PS nanoparticles dropped from the hydrogel surface. A cell homing test with the transwell model containing PS nanoparticles with diameters of 100 nm was performed in a 24-well culture plate; each well was seeded with 3.0 × 10^3^ of MCF-7 cells for overnight growth. The following protocol was the same as one mentioned above. Cells attached to TAH were measured by CellTiter-Glo (Figure 5B). The group of TAH/75% ethanol/100 nm PS nanoparticles showed the greatest attachment of cells, followed by the group of TAH/75% ethanol. Based on the same stiffness, the result showed MCF-7 preferred surfaces with higher roughness for cellular attachment. However, the numbers of attached cells were pretty low in those groups without the treatment of 75% ethanol. This confirmed the fact that proper stiffness is required for the homing of cancer cells. 

MCF-7 cells attached to the alginate hydrogel film were observed using confocal laser microscopy coupled with the post-processing of z-stacks. Starting from the first image that contained cell structure, images within the range of 15 μm were recorded layer by layer and overlapped, as shown in Figure 5D. Red fluorescence represented signals from CellTracker retained in MCF-7 cytoplasm and green ones were from polystyrene beads. The distribution of MCF-7 cells was speculated according to the analysis of the ratio of two kinds of pixels overlapped to the pixels from cells only. In the microenvironment of 4% alginate hydrogel without any further treatment, the overlapping ratio of cells to the total area of beads was 20.5%, and in the group of 75% ethanol-treated TAH, the overlapping ratio was 4.5%. Although overlapping ratios were different, this contacting phenomenon was found at the edges of the cells in both cases. For this reason, MCF-7 cells may prefer those concave structure formed between PS beads on the surface of hydrogel film for their migration and adhesion.

### 2.3. Gene Analysis

#### Physical Cues of TAH Model Regulate Stemness-Dependent Homing of Breast Cancer Cells

**Figure 6:**
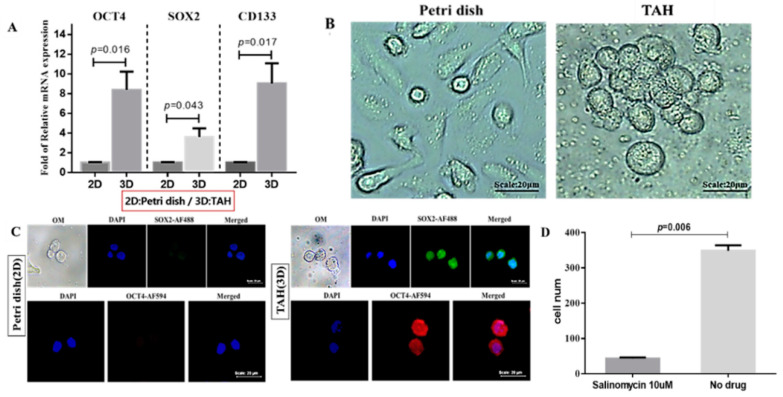
(**A**) Expression analysis of *OCT4*, *SOX2*, and *CD133* at transcription level in MDA-MB-231 cells with different culture conditions where *GAPDH* was used as the internal control, *p* value < 0.05 in all three groups. (**B**) Migratory phenotypes of MDA-MB-231 cells in petri dish (2D) and TAH (3D). (**C**) Immunofluorescence staining demonstrated that MDA-MB-231 cultured in a 3D microenvironment of TAH exhibited elevated expression levels of stemness regulators in nucleus. (**D**) Homing test of MCF-7 cells treated with salinomycin or not, *p* value = 0.006. Values shown are mean ± SD of three independent experiments.

The phenomenon of homing breast cancer cells changing from 2D to 3D in the TAH model could imply that those cells were provided with higher potential of stemness characteristics. Thus, the expression of relative biomarkers were evaluated in MDA-MB-231 through quantitative real-time PCR and immunofluorescence (IF) staining in transcriptional and cellular levels, respectively. Related genes were selected based on their putative functions in the formation of intracellular stemness, including *OCT4*, *SOX2*, and *CD133*. Independent triplicates of cells cultured on petri dish and TAH films were performed and their RNAs were freshly extracted and analyzed by qPCR. The result showed the transcription level of all three genes were significantly higher in cells grown in the 3D environment of TAH, as shown in Figure 6A. To analyze the influence of local environment on the morphology of homed cells, their phenotypes were thus determined by optical microscopy (Figure 6). MDA-MB-231 cells exhibited only circular morphology in the microenvironment derived by TAH, but cells grown on petri dishes showed ordinary morphology, with a flat and elongated shape. Then, the cellular level of stemness markers like Oct4 or Sox2 in vivo were examined by antibody-mediated IF staining, as shown in Figure 6C. The cellular translational level of both genes were much higher in TAH groups. Also, the fluorescence signals of Sox2 (green) and Oct4 (red) were highly overlapped with nucleus (DAPI in blue), where both stemness regulators triggered the transformation process from ordinary cancer cells to CSCs. For the further testing of stemness of cells attached on hydrogel film, an inhibitor of stemness marker *STAT3* was introduced into the homing test [70]. Each well was seeded with 3.0 × 10^3^ of MCF-7 cells then 10μM of salinomycin was added in for another 24-h incubation. The number of cells attached on TAH was measured by CellTiter-Glo (Figure 6D). By the abolishing of *STAT3* signaling pathway using salinomycin, homed cells were greatly decreased to the level about 15% compared to the control group, indicating these cells acquired the stemness feature. In short, all evidence, including the changing of stemness marker in molecular levels and morphology, supported that the TAH model is a novel platform suitable for the growth of stem cells.

## 3. Discussion

To build a three-dimensional model with microenvironments mimicking those of a human body will accelerate research progress in cancer biology. The biophysical factors of microenvironment are effective regulators in cellular functions and morphogenesis of tissues or organs. However, most previous research depends on the traditional culturing system based on a 2D surface which is inadequate to represent the circumambient microenvironment of solid tumors in vivo. Also, partial findings concluded in 2D systems are not applicable to animal models. Biological divergences exist in these two distinct systems, such as in signal transduction, cellular morphology, and the biophysical mechanisms of homing [39,71]. The TAH model based on a 3D microenvironment shows many advantages including simple manufacturing process without any particular instrument or crosslinker and highly reproducible hydrogel films. Alginate hydrogel used in TAH adopts an ordinary curing process: dipping 4% sodium alginate coated on the bottom of transwell insert in the solution of 0.1 M SrCl_2_ for four hours of solidification. Since the maximum distance between the surface of culturing plate and TAH film is about 300 micrometers, this spatial hindrance is competent to simulate the mechanism of microenvironment trigging the homing phenomenon of tumor cells in vivo (Figure 3).

Physical stimulations like stiffness of extracellular matrix and osmotic pressure have tremendous impacts on cellular behavior [72,73]. Through the controlling of manufactured parameters of alginate hydrogel films, physical factors like stiffness, porosity, and topology can be extraordinarily simulated. By the detection of homing phenomenon of breast cancer cells, a novel platform TAH, has been developed and characterized, which is provided with biophysical particularities of extracellular matrix. In addition, cellular adhesion and homing behavior can be controlled by adjusting the physical properties of alginate hydrogel films. This model illustrates that hydrogel is highly structural similar to ECM in vivo, so the adjustable physical factors of that can simulate the optimal matrix in favor of various kinds of tumor cells. This niche sheds light on the investigation of their specific behaviors, like migration or gene expression.

In the FBS loading test, higher concentration of FBS increase the ability of migration and the adhesion of MDA-MB-231 cells, especially in the group of 60% FBS, which indicates that the porous structure of hydrogel films causes the diffusion of nutrient supporting cell growth. By adjusting the concentration of alginate solution, the porosity of hydrogel film is easily controlled very close to that of the capillary. This superiority of TAH can promote research of the extravasation and metastasis mechanisms in different types of cancer. Cells detached from solid tumor are capable of microvascular penetration and enter the circulatory system for metastasis. When reaching favorable tissues or organs, extravasation will occur and a new tumor will colonize at distant sites. The concentration of TNF-α and immunoglobulin-like cell adhesion molecules like ICAM-1 and VCAM-1 will trigger the chemotaxis which is directly related to the metastasis of tumor cells by vascular penetration [74,75]. It is well known as the permeability of a microvessel is between 10 to 60 kD. This parameter of hydrogel is examined by measuring diffusion rates of three compounds with different molecular weight that represent different diameters in size. R6G with the diameter of 1.064 nm can penetrate in all concentrations of hydrogel films, and dextran-FITC with a diameter of 2.246 nm shows similar diffusion in hydrogel below 4%. This result shows that the pore distribution should be homogeneous in the hydrogel. However, the diffusive phenomenon is not observed in any group of hydrogels tested with Rhodamine B with diameter of 3.645 nm. Thus, the pore size of 2% alginate hydrogel should be less than 3.6 nm. The porosity of 4% alginate hydrogel film shows similar permeability to that of microvessels in humans. Based on the permeability and unique structural design of TAH, the chemotaxis of tumor cells in microenvironment can be assayed easily by loading the specific growth factor or seeding feeder cells in the top space of the transwell insert.

Stiffness is a crucial parameter of a microenvironment, which influences cellular infiltration and matrix remodeling in the process of cell proliferation [26,76]. This concept is elucidated by using ethanol as a dehydration agent which directly and physically reacts with hydrogel to elevate its stiffness. The adhesive and homing phenomena of MDA-MB-231 is promoted with increasing stiffness, as shown in Figure 5B–D. The stiffness of 75% ethanol treated hydrogel is most preferred and its compression modulus is about 1800 kPa which is close to that of the cartilage and bone marrow in humans. This observation is highly correlated to the clinical outcomes of bone metastasis in breast cancer patients. Furthermore, both MDA-MB-231 and MCF-7 show increasing numbers of adhesive cells, 4- to 7-folds respectively, in the 75% ethanol treated TAH. This observation is consistent with previous studies combining clinical samples and metastatic models made with polyacrylamide. [77,78]. Both studies indicate that stiffer ECM will promote the invasion, migration, and metastasis of breast cancer cells.

Cell adhesion and homing behavior are affected by the surficial nano-topological structure. The number of homing MDA-MB-231 cells is about two-fold in 4% alginate mixed with 100 nm PS nanoparticles than in alginate alone. In addition, in the combination of PS nanoparticles and 75% ethanol group, the number of homing cells can increase about 8- to 10-folds. The result supports that the nano-topological structure may influence cellular localization and migration, especially in motility, which can be enhanced and controlled directly by the local density and anisotropy derived from microenvironments. Different from random cell migration on isotropic nanotopography, directional migration can be achieved on anisotropy where the cell extends and retracts lamellipodia preferentially along the long axis. Directional migration (homing) can be regulated by the polarization of the microtubule organizing centers [79]; its speed is dependent on the width and depth of underlying nanogratings [79,80]. On the other hand, cell behavior is affected by the hydrophobicity of hydrogel [81], which is demonstrated respectively in the migration test by a mineral oil–hydrogel mixture or 95% ethanol treated hydrogel. Both results represent that in more hydrophobic TAH, the number of attached MDA-MB-231 cells obviously decrease (see Figure S1). Integrin is a transmembrane molecule that plays a major role in mechanotransduction. It helps cells to perceive and respond to the physical parameters of ECM. The biophysical properties of ECM, such as stiffness and roughness, mainly regulate cellular behavior like migration and adhesion through integrin, which is able to convert the signal of physical contacts to biological signals leading to the following intracellular signal transduction [82,83,84]. Previous studies have shown that the expression of integrin α6/α6β1 is upregulated with increasing ECM stiffness, which leads to more focal contacts between cells and microenvironments to increase cell migrating ability [28,85,86,87]. For this reason, it is assumed that the expression level of integrin α6 of breast cancer cells is directly regulated by physical parameters like the stiffness and roughness of TAH, which is reflected in the number of homing cells. Further studies will be carried out on the relationship between the cell behavior and different kinds of intracellular signal molecules induced by the physical properties of ECM.

Hydrogels with different surficial topologies can significantly affect the spreading process of mesenchymal stem cells and further modify their differentiation potential and propensities into destination cell types. Thus, through changing the mechanical factors supporting growth in the microenvironment, like stiffness and topology, it is practicable to regulate the fate of stem cells [45,88]. Due to both parameters being well controlled in TAH, the stemness gene expression in homing MDA-MB-231 are analyzed. In comparison with ordinary 2D culture, cells migrated from plate surface to TAH film are characterized as CSC with elevated expressions of *OCT4*, *SOX2*, and *CD133* by 4- to 8-folds, respectively. Also, their phenotypes are confirmed by immunofluorescence staining where the stemness markers Oct4 and Sox2 are overexpressed in the nucleus of homing cells. In the microscopic observation, their morphologies are round or clustered, as shown in Figure 6B. When the stemness marker is suppressed by an inhibitor like salinomycin, the homing phenomenon will greatly decline due to the blocking of signal pathway involved in stemness transformation [89,90]. All evidence indicates that TAH represents the authenticity of the 3D microenvironment used in cancer research where the interaction between of cells and matrix is systematic and reproducible. This novel model can be further applied in the evaluation of the metastasis potential of tumor cells isolated from tissues or blood, like circulating tumor cells (CTC).

Several methodologies have been used in CSC culturing, such as scaffold-supported 3D systems or spheroid culturing by an ultra-low attachment plate [91,92,93,94]. However, most of them are costly, time-consuming, labor intensive, and very sensitive to environmental parameters [95]. Also, culturing materials and medium additives, like Matrigel and particular growth factors, need to be prepared freshly [96]. These issues are still waiting to be overcome by an ideal platform. In this study, we have demonstrated the advantage of TAH in the separation and enrichment of CSCs from adherent breast cancer cells in an economic design. Not only is the operation the same as traditional cell culture, but the materials and conditions are not limited by temperature or specialized medium. TAH takes less than 24 h for gathering homing CSC, which is the most effective platform to date. The shelf-life of TAH at room temperature is at least for four weeks when immersed in a strontium solution (Table 2).

This study describes a feasible 3D-modeling strategy of biocompatible material with adjustable physical properties. Although the complexity of tumor microenvironment is not able to be simulated perfectly in vitro, some preliminary and decisive features of homing cues in breast cancer cells have been examined. Biophysical effectors like stiffness, porosity, and topology are crucial parameters in CSC homing.

## 4. Materials and Methods

### 4.1. Preparation of Materials for Hydrogel Curing

**Figure 7:**
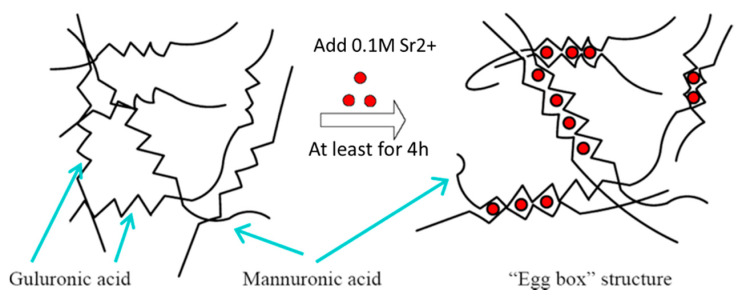
Curing of alginate hydrogel with the addition of strontium or calcium ions and the formation of an “Egg box” structure. (Modified from Wan et al.).

The main component of hydrogel was sodium saltofalginic acid which was purchased from Sigma-Aldrich (180947, St. Louis, MO, USA). The powder of sodium alginate was dissolved in distilled water (*w*/*v*) then the solution was kept at 75 °C for at least 12 h stirring at 1200 r.p.m to make all powders dissolved completely. The concentration of 2%, 4% and 6% of sodium alginate solution was obtained respectively. For the solidification of alginate hydrogel (curing), crosslinking was needed to be carried out by using bivalent ions of alkaline earth metals (e.g., magnesium, calcium, strontium and barium). Strontium ion was selected as the curing agent in this study for the capability to reaching higher stiffness, thus strontium chloride (93-3806, Strem Chemical) solution was prepared from SrCl_2_ powder dissolved in distilled water to make the final concentration of 0.1 M. This solution was then filtered sterile by a membrane with a pore size of 0.22 μm. Both solution was stored at 4 °C for further use in curing process. Alginate hydrogel was crosslinked by 0.1 M SrCl_2_ for at least 4 h, which was described in Section 2.1. Figure 7 shows the egg-box structure of alginate after curing [102].

### 4.2. Morphological Characteristics of Alginate Hydrogel Films

#### 4.2.1. Scanning Electron Microscope (SEM)

The morphological characteristics of alginate hydrogel films were observed by scanning electron microscopy. The hydrogel samples were lyophilized and mounted on a specimen stub, which was placed in a sputter coater for gold coating under vacuum condition. A layer of gold with a thickness of 100 Å was coated homogeneously using an apparatus (ohmiker-50B, M & R Technology, Hsinchu, Taiwan). The samples were examined with a field emission scanning electron microscope (JSM-7610F, JEOL, Tokyo, Japan) to obtain images using an accelerating voltage of 15 kV.

#### 4.2.2. In-Situ SEM Scanning of Liquid Samples

In this scanning, hydrogel samples were hermetically sealed in a membrane chip for maintaining in a liquid-containing state with FlowView Aquarius Starter Kit (FlowVIEW Tek, Hsinchu, Taiwan). The thin layer of membrane in this kit had decent conductivity; as such, the hydrogel sample did not need any metal coating before imaging. After sample loading, the whole kit was sent into the field emission scanning microscope for image acquisition at 15 kV. The images were processed and analyzed by Flow AOI (FlowVIEW Tek, Hsinchu, Taiwan).

### 4.3. Mechanical Compression Test

For the purpose of measuring compressive modulus, sodium alginate was cured in a cylinder mold to form a sample for mechanical testing. These cylindrical hydrogels had a diameter of 10 mm and a height of 10 mm. Strain-stress tests were carried out using a material testing machine (TA-ELECTROFORCE 3200, TA instruments, New Castle, DE, USA). The strain-stress curve was obtained through a pressure sensor which was functionalized via squeezing of the sample to the half of its original height at a speed of 0.15 mm per second. By analyzing the strain-stress curve, the macro compressive modulus could be estimated.

Compression modulus, E ($E=\frac{\sigma }{\varepsilon }$).

### 4.4. Cell Culture

MCF-7 human breast adenocarcinoma cell line (ATCC^®^ HTB-22™) and MDA-MB-231 human breast adenocarcinoma cell line (ATCC^®^ HTB-26™) were cultured in Dulbecco’s Modified Eagle Medium (DMEM; Corning Inc., NY, USA) containing 10% of FBS (Corning) and 1% penicillin/streptomycin (Pen-Strep; Thermo Fisher Scientific, Waltham, MA, USA). The human normal epithelial cell line MCF-10A (ATCC^®^ CRL-10317™) was maintained in DMEM and Ham’s F12 medium (DMEM/F12; Corning) supplemented with hydrocortisone (0.5 μg/mL), insulin (10 μg/mL), EGF (20 ng/mL), 5% horse serum (all from Thermo Fisher Scientific), and 1% Pan-Strip. The cells were maintained at 37 °C with 5% (*v*/*v*) CO_2_ in a humidified chamber. For the purpose of cell seeding, trypsinization and cell counting were carried out by 0.05% trypsin-EDTA (Corning) and a hemocytometer. In the homing test of TAH, 5 mM of SrCl_2_ (final conc.) was added into the medium to avoid the disassociation of the alginate hydrogel. In the stemness inhibition test, 10 mM stock solution was prepared by salinomycin powder (HY-15597, Med Chem Express, Monmouth Junction, NJ, USA) dissolved in DMSO. The final concentration of salinomycin diluted by culture medium was 10 μM.

### 4.5. Nucleus Localization of Cells Attached on TAH Using Fluorescence Dye

MDA-MB-231 seeded and grown onto glass coverslips or overnight homed onto TAH were fixed by 4% paraformaldehyde for 30 min. To delineate the nuclear morphology, cell nucleus dye-DAPI (4′, 6-diamidino-2-phenylindole, Sigma-Aldrich) was diluted in a ratio of 1:1000 in PBS for staining. The fluorescent-stained cells were examined with the Olympus IX71 fluorescence microscope (Olympus Taiwan Corp.).

### 4.6. Determination of The Number of Viable Cells

The viability of the MCF-10A, MCF-7, and MDA-MB-231 cells was measured using CellTiter-Glo^®^ Luminescent cell activity assay (Promega, WI, USA) which is based on the quantitation of the ATP released from live cells. The lowest detection limit of this assay is 10 cells. For measuring the viability of cells attached on hydrogel film, the film was pushed from the side by tweezers to separate it from the transwell membrane. The hydrogel film containing homing cells was placed into a well of white opaque 96-well microplates then 100 μL of luminescent substrate was added in for incubation for 10 min at room temperature. Luminescence was measured by GloMax^®^ navigator microplate luminometer (Promega, WI, USA). All TAH migration tests were carried out in triplicates with a standard curve made from known cell numbers of MCF-10A, MCF-7 or MDA-MB-231 to approximately calculate live cell number which was expressed as the mean ± standard deviation.

### 4.7. Attenuated Total Reflection Fourier Transform Infrared (FTIR) Spectroscopy

Alginate hydrogel before and after surface modification were obtained as 1 mm thick films and analyzed by FTIR (FT/IR 4200, Jasco) with transmittance mode. The scanning was performed based on the typical IR absorption range for covalent bonds, which was from 4000 to 650 cm^−1^ with 2 cm^−1^ resolution (Paragon 1000, Perkin-Elmer, IL, USA).

### 4.8. Laser-Scanning Confocal Microscopy Analysis

This experiment was only carried out with the TAH model. The breast cancer cell line MCF-7 was pre-stained with CellTracker^TM^ Red CMTPX dye (C34552; Thermo Fisher Scientific) according to manufacturer’s protocol, and then twenty to thirty thousand cells were seeded in each well on 24-well plate. Alginate hydrogel and 300 nm PS nanoparticles which emitted FITC fluorescence were doped in the ratio of 5 to 1. After the overnight migration test of TAH, hydrogel films were separated from the membrane of transwell, as mentioned in Section 4.6. Images were taken through the confocal laser scanning microscope (LSM-780, Zeiss; Carl Zeiss AG, Oberkochen, Germany) and processed with ZEN Blue version 2.3 (Zeiss). The cell covering area was measured by Image J software version 6.0 (Image J; National Institutes of Health, Bethesda, MD, USA)

### 4.9. RNA Isolation and Real-Time PCR (qRT-PCR)

The total RNA was isolated from exponentially growing cells on TAH films or a culture plate according to manufacturer’s manual (RNeasy Mini Kit, Valencia, CA, USA). Real-time PCR was performed as described previously [103] with slight modifications: Gene-specific primers were designed to amplify 150 to 200 nucleotide fragments of target genes where *GAPDH* as a control group (see Table 3). The specificity of primer sets was confirmed by standard PCR using total genomic DNA of MDA-MB-231 cells as a template. cDNA was synthesized from 5 μg of total RNA by using Reverse Transcription Kit II (SMOBIO) according to the manufacturer’s instructions. The final reaction volume was 20 μL. The qRT-PCR was performed in 96-well optical reaction plates (Applied Biosystems, Foster City, CA, USA), and each well contained the following mixture: cDNA, 1 μL; 5 nM forward primer, 1 μL; 5 nM reverse primer, 1 μL; ddH_2_O, 7 μL; SensiFAST ™ SYBR^®^ Hi-ROX Kit (Bioline) 10 μL. The qRT-PCR was carried out in a StepOnePlus Real-Time PCR system (Life Technologies) and the thermal cycling conditions were as follows: 95 °C for 2 min; 40 cycles of 95 °C/10 s, 60 °C/10 s, and 72 °C/10 s. The data were analyzed by StepOne Software v2.3 program (Life Technologies, Carlsbad, CA, USA) where the threshold and the *C_t_* value were determined with downstream data analysis performed with Microsoft Excel. Reactions with no template were used as negative controls. The expression of target genes was normalized to *GAPDH* expression which was assumed to be constitutive in MDA-MB-231.

### 4.10. Immunofluorescence Staining for Stem Cell Markers

MDA-MB-231 seeded onto coverslips or homing on TAH for 24h were fixed by 4% paraformaldehyde for 1.5 h. Blocking was carried out with BSA-PBST (BSA: 1%, PBST: phosphate buffered saline with 0.01% tween 20, *v*/*v*) at room temperature for 1 h. PBST was used for washing and in the dilution of anti-Oct4 (1:200; ab18976, Abcam), anti-Sox2 (1:200; D1C7J, Cell Signaling), Alexa Fluor 488, and Alexa Fluor 594-conjugated donkey anti rabbit secondary antibodies (both 1:200; R37118 and R37119, Thermo Fisher Scientific). All antibody-antigen reactions were carried out at room temperature for at least 1 h. DAPI was diluted as 1:1000 in PBST for nucleus staining at room temperature for 15 min after removing excess secondary antibodies with PBST washing. Immunofluorescence images were obtained as described in Section 4.5.

### 4.11. Statistical Analysis

All experiments were performed in triplicate. All data are expressed as the mean ± standard deviation. The statistical analysis of differences was performed using t test of variance using Graph Pad Prism (version 6; GraphPad Inc., San Diego, CA, USA). The *p* < 0.05 was considered to indicate a statistically significant difference. Statistical significance is presented in the figures in the following way: * *p* < 0.05, ** *p* < 0.01 and *** *p* < 0.001, **** *p* < 0.0001.

## 5. Conclusions

A novel model of the critical step in metastasis in vivo—spatial drifting—is elucidated by the TAH model which provides a better simulation system in vitro for representing the homing phenomenon of cells detached from solid tumors. Furthermore, the discrepancy of several biological mechanisms like signal transduction, morphology, and migration between TAH and traditional scratching assay can be elucidated. For example, the *STAT3* signaling pathway is considered to be involved in stromal cell remodeling and carcinogenic reprogramming to promote the formation of ecological microenvironment before metastasis [104,105]. The signal pathway involved and participators of integrin-*STAT3* signaling can be scrutinized by this platform. Also, the forming of invadopodia and the invasion of tumor cells may be further investigated by the TAH model. The concept of tissue chips can be achieved via seeding stromal cells like osteoclast, hepatocyte or fibroblast in the top space of TAH, which will be used for screening the biomarkers of secretory factors released by stromal cells that promote the homing process. The external environmental factors like hypoxia can be also analyzed in TAH model. Additionally, our platform can be incorporated with microscale 3D printing technology using composite materials for the production of bone marrow or the scaffold of liver tissue in vitro. This tissue-based stroma will more precisely mimic the native microenvironment of solid tumor and be applied in monitoring the dynamic change and heterogeneity of tumors in clinical aspects. Correlations between TAH study in vitro and clinical outcomes will be further established. The ultimate goal of this pioneer 3D model is expected to predict clinical trial results and be used for assisting the guideline of treatment, which will emphasize its significance in cancer research and the development of new antitumor drugs.

## Figures and Tables

**Table 1:** Diffusion Coefficients of Fluorescent Compounds with Different Molecular Weights in TAH.

Dyes		Diffusion Coefficient (mm^2^/s) ± SEM		
	kD	2% TAH	4% TAH	6% TAH
**R6G**	479	6.20 × 10^−5^ ± 3.48 × 10^−6^	2.58 × 10^−5^ ± 1.43 × 10^−6^	2.40 × 10^−5^ ± 1.34 × 10^−6^
**FITC–Dextran**	20,000	1.96 × 10^−5^ ± 1.26 × 10^−6^	1.72 × 10^−5^ ± 7.79 × 10^−7^	1.19 × 10^−5^ ± 7.76 × 10^−7^
**Rhodamine B**	70,000	6.28 × 10^−7^ ± 2.35 × 10^−7^	3.90 × 10^−7^ ± 3.06 × 10^−7^	8.48 × 10^−7^ ± 2.33 × 10^−7^

**Table 2:** Comparison of Enrichment Technology for Cancer Stem Cell.

Technology	Cost	Time Required for a Culture	Operational Complexity	Strengths	Weaknesses
TAH	Very low	1 day	simple	Simple procedure; Microenvironment mimicking	Not reusable
3D culture in ultra-low attachment (ULA) plates	High	10–14 days	simple	Ready to use; long-term storage	Not reusable, costly, heterogeneous size [91,92,93]
Hanging drop	Low	5–7 days	Labor-intensive	Controllable sphere size, High-throughput production	specific cultureware, short-term culturing, difficult to replace medium [97,98,99]
Gels for 3D cultures—e.g., Matrigel	High	2–3 days	Labor-intensive	Microenvironment mimicking	Costly, temperature sensitive, complicated operation, difficulty in cell isolation [50,100,101]

**Table 3:** Primers for *CD133*, *OCT4*, *SOX2* and *GAPDH* Genes.

Gene	Primer Sequences	
Target	Forward Primer	Reverse Primer
*CD133*	5′-TCCACAGAAATTTACCTACATTGG-3′	5′-CAGCAGAGAGCAGATGACCA-3′
*OCT4*	5′-CTTGCTGCAGAAGTGGGTGGAGGAA-3	5′-CTGCAGTGTGGGTTTCGGGCA-3′
*SOX2*	5′-AAATGGGAGGGGTGCAAAAGAGGAG-3′	5′-CAGCTGTCATTTGCTGTGGGTGATG-3′
*GAPDH*	5′-TGAAGGTCGGAGTCAACGGATT-3′	5′-CCTGGAAGATGGTGATGGGATT-3′

## Supplementary Materials

The following are available online at https://www.mdpi.com/2072-6694/12/8/2176/s1, Figure S1: Effects of hydrophobic hydrogel on cell migration.
