# Supplementary material for: Physical Cues in the Microenvironment Regulate Stemness-Dependent Homing of Breast Cancer Cells
Source: Cancers (Basel). 2020 Aug 5;12(8):2176. doi: 10.3390/cancers12082176 (PMC7464848; doi:10.3390/cancers12082176)
Supplement: Supplementary file 1 [file cancers-12-02176-s001.pdf]

Physical cues in microenvironment regulate stemness-dependent homing of breast cancer cells

Hsueh-Yao Chu, Yin-Ju Chen, Chun-Jieh Hsu, Yang-Wei Liu, Jeng-Fong Chiou, Long-Sheng Lu, and Fan-Gang Tseng

TAH surface modification with mineral oil and 95% ethanol would hinder cell adherence

The surface modification of hydrogel film was carried out via mixing mineral oil into sodium alginate by the ratio of 1:9 (v/v). Then the mixture was ultrasonificated for 3 minutes where strong dispersion occurred due to continuous shearing force and enormous impact between two phases, resulted in countless water-in-oil micelles. The comparison of before and after ultrasonification treatment is shown in figure S1A. Those micelles were immediately solidized by strontium ions described in methods. Both pure alginate-constituted and mineral oil-blended films were examined by a scanning electron microscope. Figure S1B shows their physical topologies respectively: hydrogel films without any treatment retained smooth surface but lots of protruding nanoparticles were found in the hydrogel treated by emulsification of oil droplets. Their sizes were among 800 to 1500 in diameters. The result proves that the surface morphology can be altered effectively by the above method. Furthermore, in the migration test of TAH model, the number of attached MDA-MB-231 in the group of hydrogel film containing mineral oil was less than that of standard hydrogel, as shown in Figure S1C. Although complicated surface structure caused by micelles is favored in cell migration and attachment, the hydrophobicity of mineral oil may greatly affect the contact of cells to hydrogel film in their migrating process. Furthermore, the hydrophobicity of hydrogel film was also caused by the treatment of 95% ethanol overnight. The number of cells attached on it was examined by a fluorescence microscopy and ATP-based assay. Figure S1D shows that only few cells distribute on dehydrated TAH film, as well as their ATP activity was much lower than the group of hydrogel without any ethanol treatment, shown in S1E. Both experiments prove that the hydrophobicity may affect the behavior of cell migration.

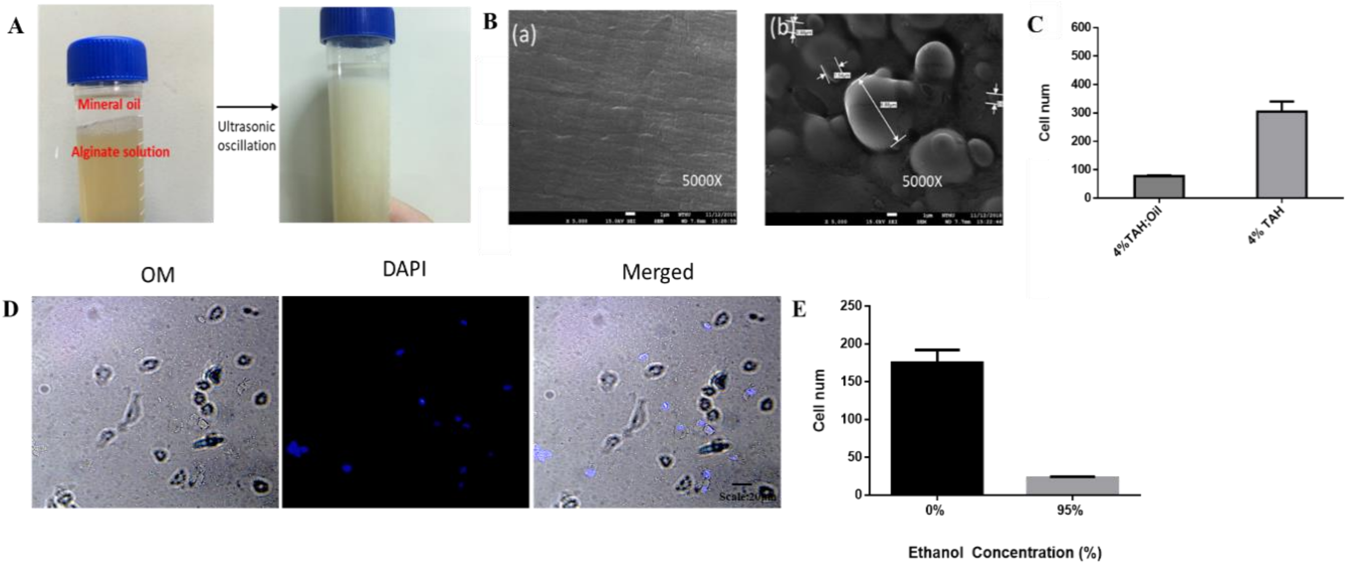

Figure S1. Effects of hydrophobic hydrogel on cell migration. (A) Emulsification of mineral oil in sodium alginate solution processed by ultrasonification. (B) SEM images of (a) standard TAH and (b) mineral oil-contained TAH surfaces. (C) The comparison of migrated MDA-MB-231 cell number in standard and mineral oil-contained TAH models. (D) Images of cell attached on hydrogel films treated with 95% ethanol. Nucleus were stained by DAPI and observed by fluorescence microscopy with the magnification of 100x. The scale bar is 20um. (E) The cell number of MDA-MB-231 migrated to alginate hydrogel films pretreated with 0% and 95 % (v/v) of ethanol, respectively.
